# Sources of Information and Behavioral Patterns in Online Health Forums: Observational Study

**DOI:** 10.2196/jmir.2875

**Published:** 2014-01-14

**Authors:** Fabian Sudau, Tim Friede, Jens Grabowski, Janka Koschack, Philip Makedonski, Wolfgang Himmel

**Affiliations:** ^1^Institute of Computer ScienceGeorg-August-University GöttingenGöttingenGermany; ^2^Department of Medical StatisticsUniversity Medical Center GöttingenGöttingenGermany; ^3^Department of General PracticeUniversity Medical Center GöttingenGöttingenGermany

**Keywords:** Internet utilization, information dissemination, data mining, social media, social networks, multiple sclerosis, CCSVI

## Abstract

**Background:**

Increasing numbers of patients are raising their voice in online forums. This shift is welcome as an act of patient autonomy, reflected in the term “expert patient”. At the same time, there is considerable concern that patients can be easily misguided by pseudoscientific research and debate. Little is known about the sources of information used in health-related online forums, how users apply this information, and how they behave in such forums.

**Objective:**

The intent of the study was to identify (1) the sources of information used in online health-related forums, and (2) the roles and behavior of active forum visitors in introducing and disseminating this information.

**Methods:**

This observational study used the largest German multiple sclerosis (MS) online forum as a database, analyzing the user debate about the recently proposed and controversial Chronic Cerebrospinal Venous Insufficiency (CCSVI) hypothesis. After extracting all posts and then filtering relevant CCSVI posts between 01 January 2008 and 17 August 2012, we first identified hyperlinks to scientific publications and other information sources used or referenced in the posts. Employing *k*-means clustering, we then analyzed the users’ preference for sources of information and their general posting habits.

**Results:**

Of 139,912 posts from 11,997 threads, 8628 posts discussed or at least mentioned CCSVI. We detected hyperlinks pointing to CCSVI-related scientific publications in 31 posts. In contrast, 2829 different URLs were posted to the forum, most frequently referring to social media, such as YouTube or Facebook. We identified a total of 6 different roles of hyperlink posters including Social Media Fans, Organization Followers, and Balanced Source Users. Apart from the large and nonspecific residual category of the “average user”, several specific behavior patterns were identified, such as the small but relevant groups of CCSVI-Focused Responders or CCSVI Activators.

**Conclusions:**

The bulk of the observed contributions were not based on scientific results, but on various social media sources. These sources seem to contain mostly opinions and personal experience. A small group of people with distinct behavioral patterns played a core role in fuelling the discussion about CCSVI.

## Introduction

In the past few decades, we have witnessed a powerful movement toward an active, self-managing, and responsible patient, coined the “expert patient” [1,2]. A key element in this process has been unlimited access to and intelligent use of health-related information, particularly that which is widely available on websites and online forums on the Internet and in online social media [3-6]. This movement has consequences for the traditional way of information dissemination. Today, laypeople, self-support groups, patient advocates, and other stakeholders can raise their voice and can even influence both public and scientific debates. This shift is welcome as an act of patient autonomy and freedom to seek alternatives to the standard therapeutic regimens and the paternalistic doctor-patient relationship. At the same time, there is considerable concern that patients can be easily misguided by pseudoscientific research, because typically they do not have the expertise to assess the reliability of scientific information and because of their circumstances may often accept any suggested solution no matter how unlikely and unrealistic it may seem. Nettleton et al [7] call for a strictly empirical analysis to examine people’s accounts of their use of online health resources.

Most studies in this area have investigated how often people use the Internet for retrieving health information [8], how they access health information on the Internet [9], which factors are important for laypeople when using Internet resources for health issues [10], and how to assess the quality of health information for laypeople on the Internet [11]. There is another area of research that seems promising—social network analysis [12]. Health-related online communities, as one form of social network, are thought to develop their own quasi-professional knowledge of their health conditions [12] and to personalize support [13]. Following applications in marketing, research has investigated diffusion processes of successful new products with the aim of targeting “influential” members of a network [14]. In the medical area, for example, a recent study showed how a social network of parents influenced decision making on vaccination in an unfavorable manner [15]. Similar concerns about misinformation via Twitter arose about flu treatment requiring antibiotics [16].

However, we still know very little about what mechanisms of information dissemination are effective as well as what sources of information people in online forums rely on, how they form their opinions, and how they act. A better understanding of these mechanisms may help to assess their influence on laypeople and to forecast the benefits and dangers of these new forms of information dissemination and exchange.

One promising area for such research is the recently proposed Chronic Cerebrospinal Venous Insufficiency (CCSVI) hypothesis in multiple sclerosis (MS) and its repercussions in patient communities. In short, this hypothesis was first proposed by Paolo Zamboni [17], who suggested that obstruction to venous drainage in the neck and spinal cord [17], termed chronic cerebrospinal venous insufficiency, was linked to MS [18,19]. Although the association between MS and sonographic features of CCSVI is variable [18], some institutions have even begun to offer angioplasty and endovenous stenting of CCSVI, often referred to commercially as “The Liberation Procedure” [20]. The intensity of the CCSVI debate reached such a point that the Society for Interventional Radiology released a position statement regarding endovascular management of CCSVI [21]; the MS Society of Canada funded a study of the prevalence of extracranial venous narrowing, which found evidence neither for a high prevalence of CCSVI nor for its causal relationship to MS [22]. Several studies report a wave of complications following venous stenting and angioplasty [23,24]. The CCSVI hypothesis is also fiercely debated in online patient communities, such as the online forum of the German MS Society (DMSG, Deutsche Multiple Sklerose Gesellschaft) [25] and the United Kingdom’s MS Society online forum [26], as well as numerous other dedicated websites and blogs on the Internet [27,28]. It has even found its way to the popular video-sharing website YouTube [29], with more than 23,800 videos posted up to July 2013, one of them with more than 200,000 views. The CCSVI waves seem to have calmed down and some consider the hypothesis—in a retrospective view of the CCSVI hype—as a waste of valuable time, money, and intellectual energy [30]; others emphasize that the debate has stimulated the need for studies that should contribute to a better understanding of the function and role of the extracranial venous system [31].

Before we can make a statement on whether and how this multitude of information sources and opinions may contribute to the enlightenment of some participants in the debate or the confusion of others, we need to know more about the sources of information used in online health forums and how users and participants use this information, including their different roles and contribution behavior in such forums. To examine these questions, we can build on a UK study on online self-harm discussion forums [32]. Using “social networking metrics”, the authors found different types of online discussion participants and roles: the Caretaker (being always watchful, participating to some degree but not initiating many new threads in discussions), the Butterfly (logging on very frequently with quick looks around and then logging off again), the Discussant (initiating many discussion threads), and the Here for You (initiating few discussion threads but posting the most comments).

Our observational study takes advantage of free access to a large German online forum related to multiple sclerosis, with the aim of identifying (1) sources of information used in online health forums, and (2) roles and patterns of behavior of people actively engaging in the forum in introducing and disseminating this information.

## Methods

### Design

In this observational study, we extracted the content from an online health forum, using a custom implementation of a Web crawler, with the aim of collecting a large database of discussions from an online health forum. Furthermore, we used an Information Retrieval algorithm (specifically designed and implemented for this particular task) to identify a comprehensive sample of posts dealing with CCSVI.

### Database and Retrieval of Relevant Posts

**Figure 1:**
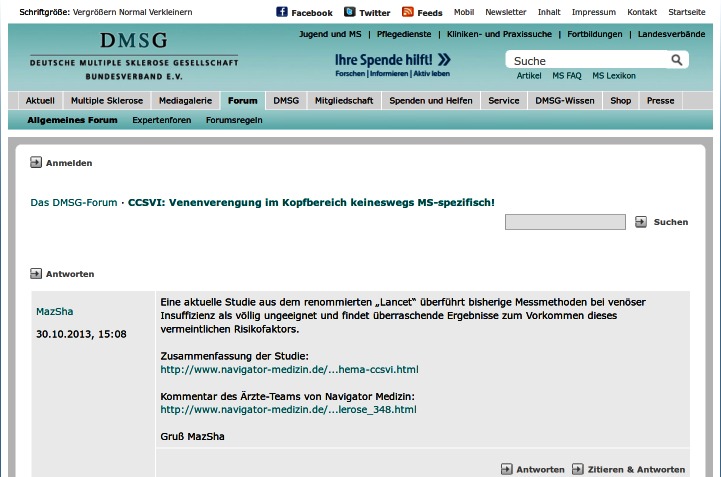
A screenshot of a forum post.

The database for the study comprised contributions posted to the online forum of the Deutsche Multiple Sklerose Gesellschaft (DMSG, German Multiple Sclerosis Society) [25]. On its website, the DMSG presents itself as a non-profit stakeholder of MS patients and their families, founded by clinical and scientific experts in MS in 1952. It is a registered charity with 16 regional branches and over 900 community contact groups. Among other things, the DMSG provides on its website two different kinds of freely accessible forums: one expert forum with time-limited chats between experts and users about different issues (eg, cognitive deficits in MS or pregnancy in MS). The other forum is unstructured, not moderated, and open for anonymous registration. It is targeted at laypeople, mostly people with MS. The forum consists of threads, which in turn contain sequences of posts. These posts can contain hyperlinks and can cite any number of previous posts. A screenshot of such a post is shown in Figure 1.

Between 01 January 2008 and 17 August 2012, all 139,912 posts from 11,997 threads were extracted. Because the forum is about MS in general, only a fraction of the extracted posts were expected to be about CCSVI. Preliminary analysis showed that the assumption of “one thread discusses one topic” does not hold in the observed forum. Instead, users tended to deviate from the original topic as time progressed. Therefore, a custom Information Retrieval algorithm was developed to classify individual posts as either relevant (“discussing CCSVI at least partially”) or irrelevant. For details on the algorithm design, training, and evaluation, see Multimedia Appendix 1. The algorithm identified 8628 posts as relevant, which yields a distinction important for further analysis steps.

### Search for Scientific Publications

Because the term “expert patient” implies intelligent use of scientific information, we aimed to assess to what degree the use of scientific sources was present in the forum. Users occasionally included hyperlinks in their posts and these links referred to content the users based their opinions on. We analyzed which of these links were defined references to scientific papers in order to get an overview of the kind of papers cited and the temporal citation patterns. Two steps were necessary for this identification process.

First, we generated a presumably exhaustive list of publications dealing with CCSVI. A citation network starting from Zamboni’s original publication and using the CiteXplore Web service was constructed [33]. CiteXplore, which is an interface to the PubMed search engine, was used due to easy accessibility of citations. These publications were then merged with a second list that was obtained by a search for “CCSVI” in the PubMed database via the Entrez interface [34]. The merging algorithm removed duplicate publications as identified by their PMID identification number. Our final publication list does not include publications that deal with CCSVI but do not include the CCSVI acronym, or had not shown up in the citation network. We assume this number of publications to be low and prefer our method over manual approaches.

Second, a program fetched every hyperlink (also those in “irrelevant” posts) from the corpus, extracted the textual content from the referenced webpage or PDF document and searched it for titles or publication IDs from the publication list. In the case of a hit, one of the authors verified whether one of the publications was indeed referenced and, if so, which one. Every match was also classified as either a direct reference or an indirect reference. An indirect reference in this context was regarded as a resource that solely discussed or explained a certain publication, not including other work based on the publication. A direct reference linked to the publication itself.

### Search for Other Web Resources Used and Their Classification

Apart from searching for scientific information sources in the posts, we also strived to identify other information sources used or referenced in the posts. In order to obtain an overview of the wide spectrum of referenced websites, we defined a classification scheme. First, we reduced every URL found in the reduced corpus to the basic domain part of the URL (ie, only “domainname.com” was used—if the URL included additional content after the domain name, such as directories, folders, webpages, file extensions, that content was removed from the URL). Second, we classified the remaining domains into the 8 classes shown in Table 1. These classes were defined based on content type and authorship provided under the respective domain. A plot was then generated showing the number of URLs from each class posted per month.

**Table 1:** Primary domain classes.

Organization	Meant in a broader sense, including foundations, associations, and unions. These are sometimes professional and often promote some kind of agenda.
Commerce	Private business selling products or services that do not include treatment.
News	Commercial news providers.
Other	Various content not fitting into the other classes.
Personal	Static content from a single person.
Scientific	Sources of scientific work and knowledge including Wikipedia. We included the latter in this class, because its reliability was established in [35]. We believe that Wikipedia, in contrast to sources from other classes, is perceived as a factual source by most of the users.
Social	Social media websites revolving around communication and user-generated content.
Health care providers	Doctors’ offices, clinics, Q&A by professionals. Not limited to Multiple Sclerosis.

### User Behavior

To characterize user behavior, we tried to identify distinct behavior patterns. Since nothing was known in advance about the behavior patterns of forum users, we employed a method of exploratory data analysis to reveal possible patterns. A clustering algorithm groups users based on their similarity according to a set of predefined features. We thus wanted to define two separate feature sets with the aim of describing two different aspects of user behavior and revealing patterns in these features through clustering. We employed the popular *k*-means clustering algorithm (originally proposed in [36]) to group the data vectors representing users together based on how close they were to each other in the Euclidean hyperspace. The algorithm was chosen due to its simplicity and widespread use. Users within one cluster were thus assumed to display similar behavioral patterns, different from those patterns prevalent in other clusters. We defined the user cluster names based on manual inspection of descriptive cluster statistics.

Two behavioral aspects in particular were analyzed in detail by separate clusterings: (1) the preference for discussed sources of information, and (2) the general contribution behavior or posting habits. In the first clustering, we focused on the hyperlinks from each of the 8 domain classes. A user was represented by a vector in 8-dimensional space: for example, a value of 3 for the 2^nd^dimension meant the user had posted 3 hyperlinks from the domain class “Organization”. The second clustering focused on 9 quantitative features describing what and how a user had posted. The features (measures) were either taken from similar approaches discussed in the literature [37] or defined according to metadata that has not been used previously. Specifically, the literature does not use measures based on the distinction between on-topic and off-topic talk and does not make use of possibly insightful metadata such as hyperlinks or citations. The features and the reasoning behind them are described in Table 2. All of them are defined over the entire contribution period.

In both cases, the *k*-means clustering algorithm was used in the form of a custom implementation. We employed a heuristic initialization step to compensate for adverse effects of bad initial centroid placement. The clustering terminated when no more cluster memberships changed. For details on the employed algorithm, see Multimedia Appendix 2.

In the first clustering, we had to compensate for different general activity levels of users because we wanted to group the users according to their information source preferences only. We divided every vector by its Euclidean norm in order to obtain unit vectors showing only “taste” (preference), but not “activity”. In the second clustering, the different features had different scales. For instance, users often showed several hundred days of activity, but the fraction of their initiated threads can by definition not exceed 1. We thus performed a *z*-score normalization of the data before the second clustering. This means we modified every feature value of every user vector as follows. First, we subtracted the feature mean (over all users) and then we divided by feature standard deviation. The *k*-means algorithm requires that the number of clusters (*k*) is specified in advance. Preliminary experiments with different values of *k* showed that *k*=6 was a good choice in both cases, judged by manual inspection of internal cluster evaluation metrics and resulting cluster sizes.

We visualized the resulting clusters in radar charts [38], also known as spider charts or kiviat diagrams. A radar chart has a “spoke” for each feature; the data length of a spoke is proportional to the magnitude of the variable for the data point relative to the maximum magnitude of the variable across all data points so that multivariate observations with an arbitrary number of variables can be displayed and compared. Star-like figures indicate normalized feature means across the members of a cluster. Although it is difficult to compare lengths of different spokes visually, striking differences as well as commonalities between clusters can be captured easily and therefore the characteristics of the different clusters are thus easily comparable. We gave names to the clusters based on manual assessment of the radar charts and the tables of Multimedia Appendix 3. The definition of these names is based on which feature values “stand out” for a given cluster.

**Table 2:** Definition of behavior features.

Measure	Definition	Rationale
Average message length (from [39])	Average post content length in characters without counting references.	The message length is an indicator of the amount of effort that is put into a post by a user and it also tells us something about the discussion style of a user. Some users prefer elaborate, essay-like contributions while others use the forum in a more conversational way.
Average number of posts per day (from [32])	Average number of posts per day that a user made.	This is the most important activity feature of a user and it also provides an insight into the selectiveness of the user. A user with a high number of posts per day over a long time period can be expected to be a frequent visitor, who makes posts regardless of outside events.
Average number of references per post	Average number of unique references that are included in a post.	The feature describes the tendency of a user to bring new sources of information to the forum and may also describe the ability to support the stance of the user with evidence.
Average number of threads per day (from [32])	Average number of different threads a user posts to per day.	While this is also an activity feature, it provides an insight into the focus of interest a user has. A low value may indicate a preference to discuss only specific topics while a high value may indicate a preference to join any sort of discussion.
Days active (from [39])	Number of days between the first post and the last one.	The feature indicates the consistency of the contribution behavior and posting habits of a user and is an important piece of context information when interpreting the other features.
Fraction of posts that were cited	Fraction of the posts that have been cited at least once.	While it can only be assumed what users try to express when they use the citation function, the feature is expected to show the tendency to provoke direct responses from other forum participants.
Fraction of relevant posts	Fraction of the posts that were classified as relevant by the Information Retrieval algorithm.	This feature is a solid indicator of the user’s interest in CCSVI^a^. While it cannot be inferred from this feature alone whether the user has a pro-CCSVI or anti-CCSVI stance, it seems plausible that users with a high interest in CCSVI believe in the hypothesis.
Fraction of initiated threads (from [37])	Fraction of the threads the user initiated based on the total number of threads the user contributed to.	This feature measures the tendency of a user to start discussions, which is often related to the introduction of new information to the forum.
Coverage of users in relevant parts per post	Number of users the user discussed CCSVI with divided by the total number of posts the user made. An uninterrupted sequence of relevant posts is regarded a single discussion. The users that co-occurred in these discussions are counted as discussion partners.	This feature can be described as the efficiency in opinion exchange about CCSVI.

^a^CCSVI: Chronic Cerebrospinal Venous Insufficiency

## Results

### Search for Scientific Publications

We detected hyperlinks pointing to CCSVI-related scientific publications in 31 posts. Multimedia Appendix 4 gives the 13 different publications referenced by the forum users. Each publication is shown in a separate area where the red star indicates the publication date and the green diamonds show dates where links to the publication were posted. Light green diamonds indicate indirect references. Interestingly, Zamboni’s original publication [17] was brought to the forum no later than two months after publication and referenced repeatedly, often indirectly. Another 4 publications in favor of the CCSVI hypothesis [40-43] were cited by September 2010. The position of the publications was identified manually. Judging from the referenced scientific publications alone and ignoring post content as well as other references, the period from July 2009 to September 2010 can thus be described as a “boom phase” of the CCSVI hypothesis in the forum. However, after September 2010, critical publications appeared and were brought to the forum. In fact, all except one of the referenced publications after September 2010 [44-50] strongly oppose CCSVI. At this time, the series of repeated references to Zamboni’s original publication stopped.

### Search for Other Web Resources Used and Their Classification

**Figure 2:**
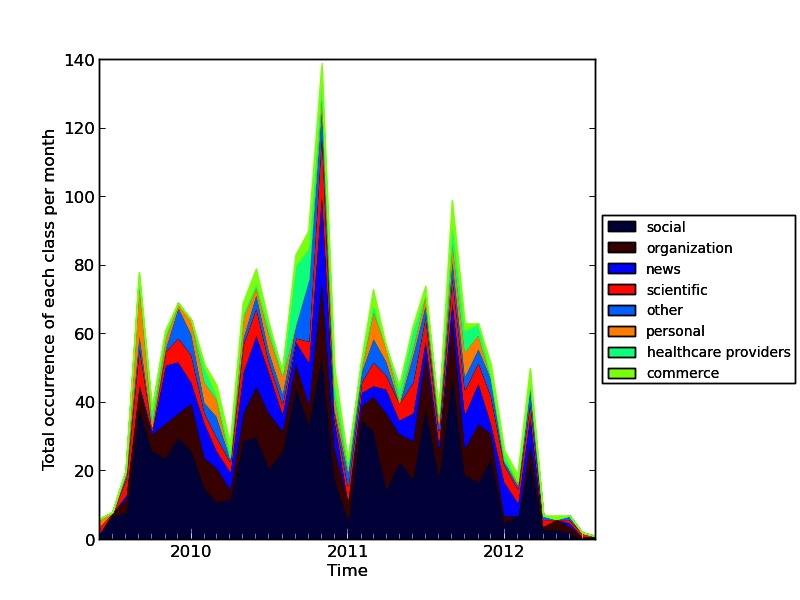
Timeline of posted hyperlinks for each domain class.

Figure 2 shows how many hyperlinks of each domain class were posted each month, sorted by overall domain class popularity. At any given point in time, social media websites were the most widely used type of Web resource. Similar to the low number of referenced scientific publications, science-based resources were generally not used very often. About half of the posted hyperlinks from the domain class “scientific” refer to Wikipedia articles. Organization-related websites and news sites were the second and third most important ones.

The large differences in the total number of posted references per month correlate roughly with the total number of relevant posts. Interestingly, the highest peak (September 2010 - November 2010) was observed when the aforementioned phases shifted. The external events causing the other significant fluctuations are not known. However, when the total number of posted references rose from a given point in time to another, the change was typically reflected in all of the domain classes, which indicates a certain echo of external events equally affecting the different types of resources. The plot also shows how quickly the topic caught on in the layperson forum and that users seemed to have lost interest in the debate, as suggested by the few references posted in 2012.

### User Behavior

**Figure 3:**
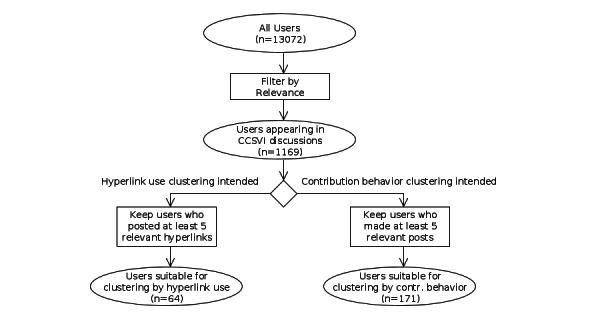
Flowchart of the sampling procedure for clusterings.

**Figure 4:**
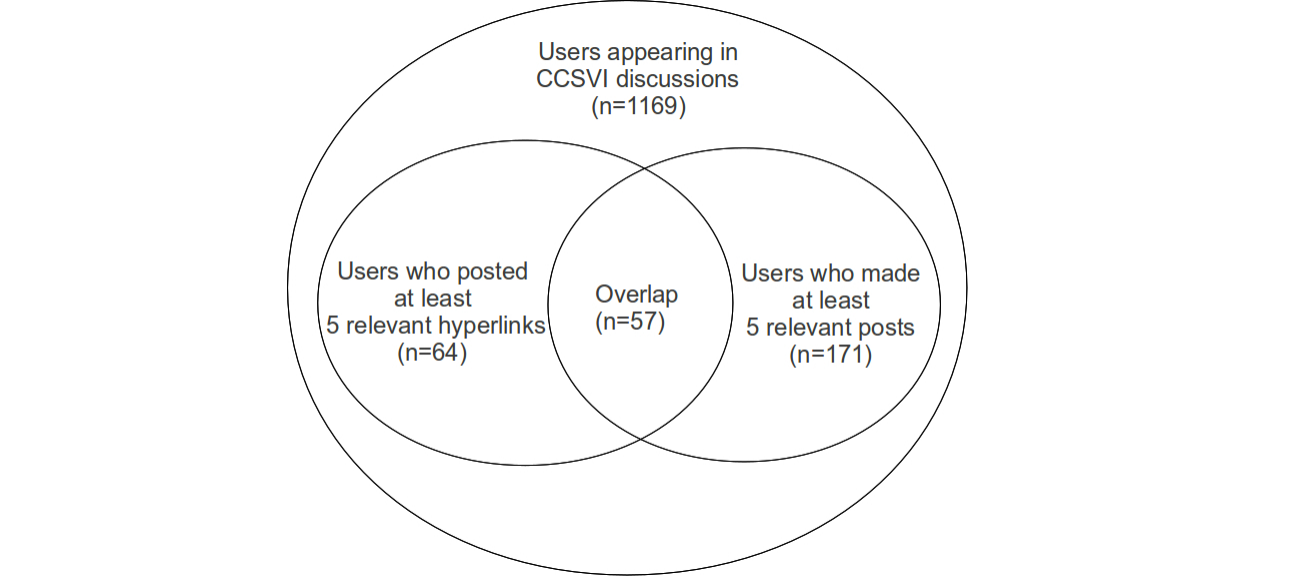
Venn diagram showing the user sets used in the clusterings.

We included only a fraction of the users in the clustering because we wanted to focus on those who took part in CCSVI discussions. Furthermore, a sufficient amount of information about each user was required. Therefore, we clustered only users who had posted at least 5 relevant hyperlinks, in the case of hyperlink use (first clustering). In the case of posting habits (second clustering), we included only users who had made at least 5 relevant posts. The filtering process is shown as a flow diagram in Figure 3 and resulting sets are shown in Figure 4. The fraction of users who were active enough for meaningful analysis is rather low, which is typical for online communities. Nearly two-thirds of the DMSG forum users posted only once.

**Figure 5:**
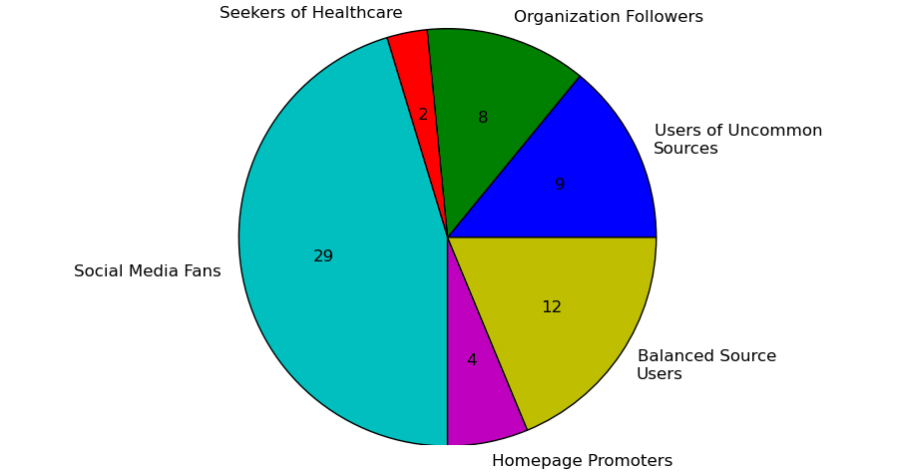
Reference use clusters with number of users in each cluster (n=64 included cases).

**Figure 6:**
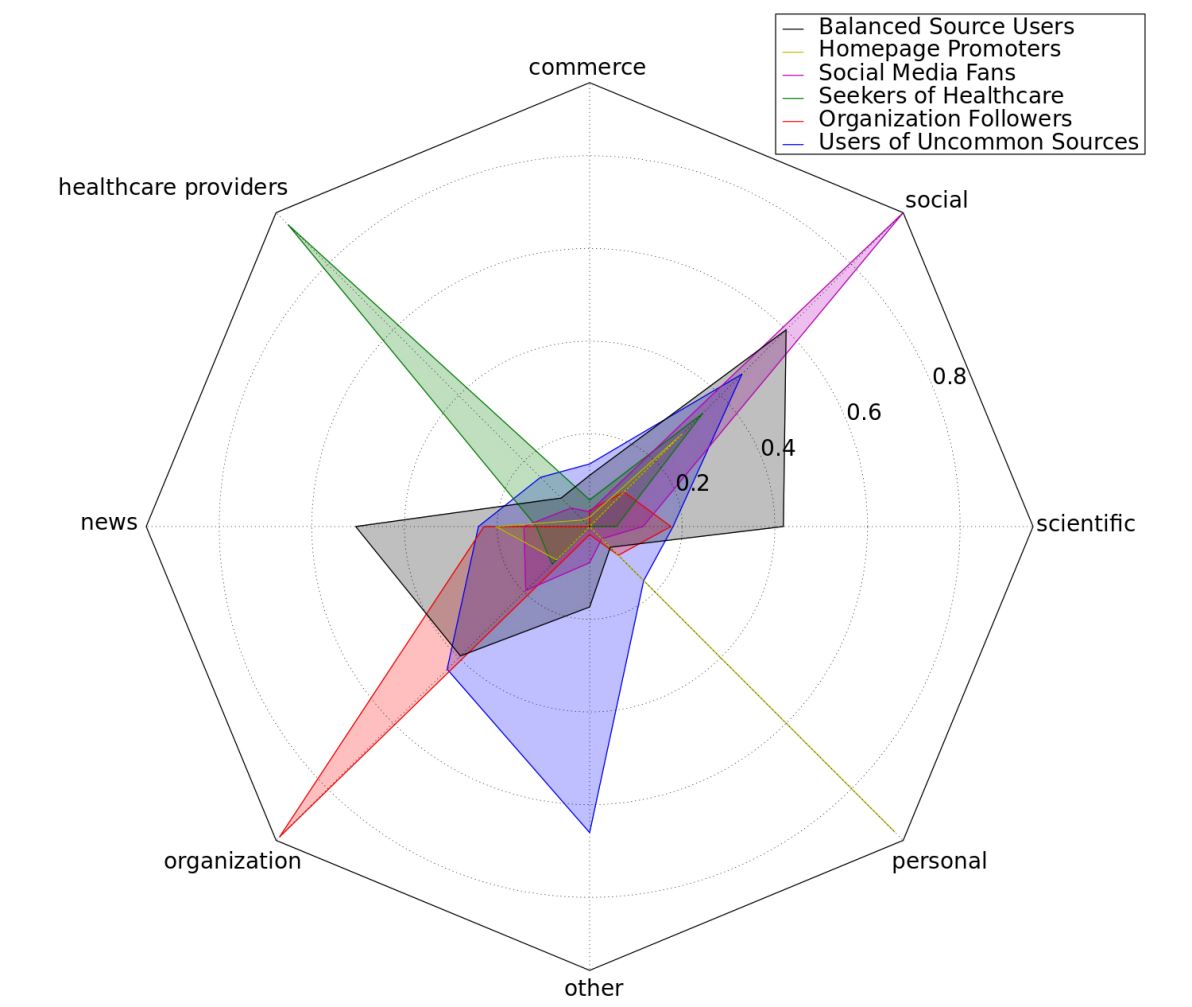
Radar chart showing aggregated domain class use of each cluster (the user vectors belonging to the cluster are summed up). Each cluster vector is a normalized to be a unit vector. The length of a spoke is proportional to the value it represents.

The first clustering of the users into 6 groups revealed clusters shown in Figure 5. Roughly half of the users (29/64) can be described as Social Media Fans. Figure 6 shows the information sources preferred by members of each cluster. Social Media Fans, for example, prefer video-sharing websites (such as YouTube.com), Facebook pages, and blogs over more traditional sources. Balanced Source Users cite sources from different classes equally often, including scientific ones. Organization Followers mainly refer to content published by organizations; we also identified a group that uses sources that do not fit well into the classification scheme. Homepage Promoters post links to websites featuring static content authored by a single person. These traditional sites already existed in the early era of the Internet. Seekers of Healthcare discuss doctors and clinics. Users of Uncommon Sources focus on religion, esoterism, complementary or alternative medicine, or unrelated resources.

**Figure 7:**
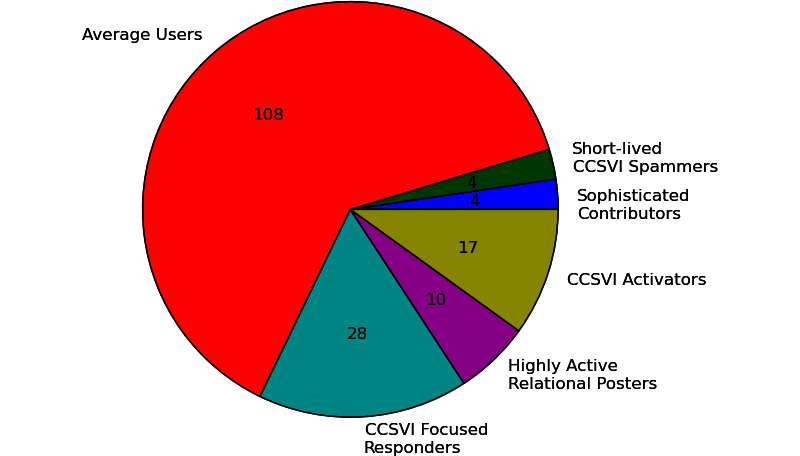
Posting behavior, according to the second clustering, with number of users in each cluster (n=171 included cases).

**Figure 8:**
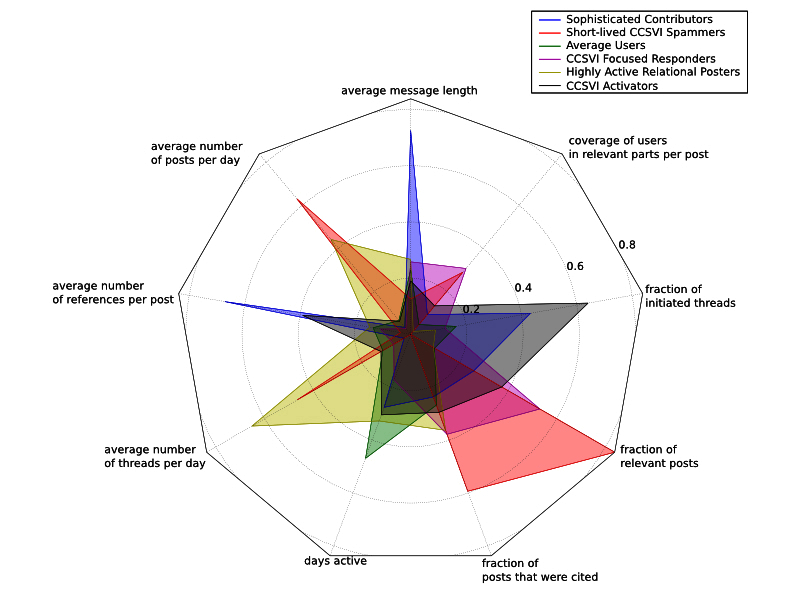
Radar chart showing feature means (overall users within a cluster) of the contribution behavior clusters. The means are min-max-normalized to a [0;1] range. The length of a spoke is proportional to the value it represents.

Clustering users, who had made at least 5 relevant posts, revealed the 6 groups shown in Figure 7. The cluster names were derived manually without a prespecified algorithm from the corresponding table in Multimedia Appendix 3 and from Figure 8, which shows the feature means normalized to a [0;1] range. About two-thirds of the users could only be described as “average”. This means that they do not stand out, but the characteristics of these users provide a baseline for comparison with the other user roles. Twenty-eight users were CCSVI-Focused Responders, who were active for less than a year on average. What defines them is the low level of posts per day, the low fraction of initiated threads, and the high fraction of CCSVI-related posts. Ten users were Highly Active Relational Posters, who show the highest level of posting activity (about 4 posts per day). They posted in lots of different threads, but rarely initiated them. Another 17 users are CCSVI Activators, who stand out due to their high fraction of initiated threads, their high percentage of CCSVI-related posts, and the fact that they included 3 times as many references as the average user. The 4 Sophisticated Contributors are known for making posts that are 3 times as long as those of average users and include 5 times more references. The remaining 4 Short-Lived CCSVI Spammers were active for a few days only and, during their short contribution period, created many posts about CCSVI. The posts were short and included few references.

## Discussion

### Summary of Main Findings

The bulk of the observed contributions were not based on scientific results, but on various social media sources. These sources seem to contain mostly opinions and personal experience. A small group of people with distinct behavioral patterns played a core role in fuelling the discussion about CCSVI, as identified by their behavior. The identification of this group of people was an unintended consequence of our exploratory analysis technique. Our identification method is behavior driven and thus provides a viable alternative to the influence-based identification of so called “opinion leaders” in forums, as discussed in [51-54].

### Meaning of the Results and Comparison With Literature

Scientific publications were brought to the forum at a “boom phase” of CCSVI discussion, followed by a phase of critical views, beginning September 2010 with the opponents of the CCSVI hypothesis getting the upper hand in the forum. Although scientific and lay discourse seem to go hand in hand, it is obvious that scientific publications and scientific sources such as Wikipedia played, in the end, only a minor role in the layperson forum. Instead, social media were the most important source of information. The nature of social media content varies, but we believe that social media are often about personal experiences and exchange of opinions. This is further illustrated by the reference use patterns we identified, such as Social Media Fans or Homepage Promoters. We would suggest characterizing the nature of this lay discourse more as an elementary discourse or an interdiscourse [55] than a special or scientific discourse.

Our 6 groups of posting behavior are based on a careful inspection of different characteristics and are similar to the participants in 5 online forums on self-harm [32]. The CCSVI-Focused Responders, characterized by a low level of posts per day, the low fraction of initiated threads, and the high fraction of CCSVI-related posts may compare to the Here For You user [32], who was very supportive in the self-harm forum. Discussants [32] may compare to our CCSVI Activators, who stand out due to their high fraction of initiated threads, their high percentage of CCSVI-related posts, and their introduction and placing of references. The Highly Active Relational Posters, who posted very actively, but rarely initiated threads, compare to Jones et al [32] Caretakers. The Short-Lived CCSVI Spammers remind us of Jones et al [32] Butterflies. The 4 Sophisticated Contributors remind us of researchers and the emergence of online expert patient groups [12]. The largest fraction of contributors could only be classified as “average”.

Only a small set of the involved users showed enough activity to be suitable for meaningful descriptions of their behavior. This is consistent with the common observation of significant participation inequality in social media. Typically, activity levels are characterized by the power law with about 1% of the users exercising the core influence on a community [56]. For example, studying the community structure of online diabetes forums, Chomutare et al [57] found very low user participation rates and suggested high levels of the few users who participated actively. About 37% (63/171) of the users participating in CCSVI discussions showed distinct patterns in their posting behavior: 28 CCSVI-Focused Responders seemed to wait for CCSVI discussions to come up and then contribute. Doing so, their post-wise efficiency of CCSVI-related information spread is the highest of all roles. This seems to be an interesting new aspect to the usually performed network analysis in online forums. In these analyses, knowledge of, and personal experience with, the disease play an important role in gaining central positions and becoming authorities [57,58]. Obviously, a good command of scientific sources of information may also be one characteristic of a group of influential figures.

The Highly Active Relational Posters are expected to be important community builders, as a substantial amount of personal “small talk” is attributed to them. Interestingly, a group of 17 people, the CCSVI Activators, played a core role in fuelling the discussion about CCSVI, because they often initiated threads about CCSVI and included many hyperlinks. While there is considerable concern that social media and Internet applications permit a minority of individuals to spread misinformation and damage useful interactions as recently discussed in the case of anti-vaccinationism [59], our results show that the CCSVI discussion in the MS forum follows the ups and downs of the scientific debate and does not promote dangerous practices or prevent novel technologies to a dangerous degree.

Some Sophisticated Contributors were identified, but these people did not participate in CCSVI discussions very often. Additionally, 4 very short-lived and CCSVI-focused accounts were identified. One possible explanation is that they were the temporarily-used alternative accounts of some users.

### Strengths and Limitations of the Study

One major advantage of this study is its observational nature. Real-world data was observed in an unobtrusive way. We analyzed a public Internet forum, which was unstructured and unmoderated, over a 3-year period of CCSVI discussion. We thus avoided self-reporting biases and artificial setups. Furthermore, we applied a Machine Learning approach in order to shed some light on the complex nature of user interaction.

However, there are several limitations. There is no demographic data available for the forum users and it is even possible that some persons used different accounts. Furthermore, before 27 August 2010, users were able to choose their aliases freely for every individual contribution. Due to the lack of a log-in mechanism, it is possible that different individuals posted under the same name.

The identification of relevant content was non-trivial and did not have 100% accuracy, which resulted in a possibly biased database. The reduction of URLs to the basic domain was a simplification. When assessing user patterns, we had to deal with small sample sizes (N=64 and N=171). The clustering approach itself relied on several assumptions. We assumed that constant behavioral patterns exist, that we defined appropriated features to describe them, and that they are linearly separable in the feature space. The interpretation of the assigned roles is subjective, but based solely on the quantitative data documented in this study.

We had to decide how to identify scientific sources of information in the posts. To be on the safe side, we accepted only the posting of URLs with a link to scientific publications as a use of scientific publications. Of course, other users may have discussed scientific publications in a rather elaborate way without posting URLs. Moreover, publications are often hidden behind a paywall, which may make posting URLs unpopular. They are also written in English, which may pose a language barrier. Our approach underestimates the discussion of scientific publications in online health forums but is highly specific in identifying the introduction of scientific publications.

Our description of participants in this online health forum was based solely on “metrics”, similar to the Jones et al study [32]. A full and reliable description of the participants’ views would require an elaborated semantic analysis of their contributions.

### Implications and Future Research

Scientific sources were by far less important than social media in the posts and forum discussions. While some of the uncovered evidence may indicate the successful propagation of scientific results into discussions among laypeople within an online health forum, scientific results represented by far a rather small fraction of the information sources that were discussed in the particular forum under study. Whether this is any indication of the rise of the “expert patient” remains the subject of further studies. Some of the participants in the forum, especially the Sophisticated Contributors, could be considered experts based on the nature of their contribution behavior and their overall behavior, with rather extensive posts often including scientific and other references. They, however, also represent only a tiny fraction and before we can draw reliable conclusions we need to conduct semantic analyses of their statements. In contrast, the majority of overall users tend to rely on social media-based sources of information, which often feature personal experiences and opinions.

The health care system can be described as a two-sided network: a network with large components linked to each other through multiple platforms so that clinicians, health care institutions, and companies can interact with patients and communities [12]. While we studied one component of this network and how it is affected by the other side of the network, further research should also focus on the opposite direction and mutual influences between the components of this two-sided network.

Our study has used some sophisticated methods for extracting information on the posting behavior in online forums to address important questions in this field. To eliminate some of the limitations of the study and to determine more precisely the role and behavior of forum contributors with regard to scientific information, a qualitative approach is needed, preferably a discourse analysis of the social exchange processes and argumentative strategies in online health forums, similar to a Canadian study of online social support forums for gamblers, in which the interaction of the participants, their common discussions, and how they constructed identities and negotiated legitimacy were analyzed [60]. We are in the midst of a change due to technology where health provision and education can increasingly benefit from using the Web, in an environment in which individuals and communities become more able and responsible for their own health and treatment [61].

## Multimedia Appendix 1

Forum information retrieval.

## Multimedia Appendix 2

*K*-means clustering.

## Multimedia Appendix 3

Clustering results data tables.

## Multimedia Appendix 4

Referenced scientific publications.

## Abbreviations

CCSVI: Chronic Cerebrospinal Venous Insufficiency
DMSG: Deutsche Multiple Sklerose Gesellschaft/ German Multiple Sclerosis Society
MS: multiple sclerosis
