# Title Correction: Sources of Information and Behavioral Patterns in Online Health Forums: Observational Study

**DOI:** 10.2196/jmir.3280

**Published:** 2014-01-31

**Authors:** Fabian Sudau, Tim Friede, Jens Grabowski, Janka Koschack, Philip Makedonski, Wolfgang Himmel

**Affiliations:** ^1^ Institute of Computer Science Georg-August-University Göttingen Göttingen Germany; ^2^ Department of Medical Statistics University Medical Center Göttingen Göttingen Germany; ^3^ Department of General Practice University Medical Center Göttingen Göttingen Germany

The authors of the paper originally entitled "Sources of Information and Behavioral Patterns in Online Health Forums: Qualitative Study" (J Med Internet Res 2014 Jan 14;16(1):e10) wish to correct the title of their publication. The subtitle "Qualitative Study" is misleading because the analysis was primarily quantitative. The title has now been changed to read "Sources of Information and Behavioral Patterns in Online Health Forums: Observational Study". This error has been corrected in the online version of the paper on the JMIR website on January 31, 2014, together with publishing this correction notice. There are no changes to the contents of the paper. A correction notice has been sent to PubMed. This was done before submission to Pubmed Central and other full-text repositories.

