# Supplementary material for: Sources of Information and Behavioral Patterns in Online Health Forums: Observational Study
Source: J Med Internet Res. 2014 Jan 14;16(1):e10. doi: 10.2196/jmir.2875 (PMC3958625; doi:10.2196/jmir.2875)
Supplement: Supplementary file 1 [file jmir_v16i1e10_app1.pdf]

Forum Information Retrieval

Preliminary analysis showed that users did not stick to the intended forum behavior of discussing exactly one topic in one thread. Instead, they deviated from the original topic over time, sometimes coming back to the original topic. In short, any topic could appear in any thread. Thus, we needed a way to decide for every individual post, whether it was relevant or not. The simplest approach is to scan every post for a set of predefined keywords. However, we assumed that the context of a post also plays a role when determining the relevance of a post. We thus defined an Information Retrieval algorithm, that extends the keyword-based approach by also taking structural (contextual) information of posts into account. (The following is mostly taken from [5].)

Reasoning behind the new Algorithm

Approaches based on linguistic features alone can not be expected to perform well. This is because:

- casual / informal language is used
- the language used varies from person to person
- posts can consist of only a few words
- from a the textual content of a comment alone, it is not always possible to say, what topic it refers to. For example, a generic comment like "I do not agree" can occur in different kinds of discussions.

The naive approach of classifying only keyword-containing posts as relevant is expected to result in a lot of false negatives. This is because comments / responses do not always repeat the discussed terms. To address these challenges, a new algorithm is proposed, that utilizes the structural features of a forum instead. The algorithm makes use of post content, post order, post author, and quotations. It relies on the following assumptions:

- the relevance of a post depends on context information from within the containing thread

- keyword occurrence is an indicator of relevance
- a post, that follows a relevant post, is likely to be relevant
- if a post cites or quotes other relevant posts, it is likely to be relevant
- a user, that often writes keyword-containing posts, is more likely to write relevant posts in general

Based on the discussed assumptions, the algorithm is defined.

Defining the Algorithm

A thread is modeled as a sequence of posts denoted by $p_0, p_1, \ldots, p_n$. The relevance of an individual post p_i is determined by calculating a real-valued relevance score using function $s()$ and comparing it to a post-independent threshold t. Thus, the classification function $f()$ can be written as:

$$f(p) = \begin{cases} \text{relevant} & \text{if } s(p) \geq t \\ \text{irrelevant} & \text{if } s(p) < t \end{cases}$$

We define $s \to [0; 1]$ and $t \in [0; 1]$. The score function $s()$ is a bounded linear combination of the factors influencing the relevance of the post as assumed above. Every feature is represented by a function ϕ, which is weighted with a constant model parameter. We define four features which results in the following score function:

$$s(p_i) = \min(k \cdot \phi_k(p_i) + c \cdot \phi_c(p_i) + f \cdot \phi_f(p_i) + u \cdot \phi_u(p_i), 1)$$

Function ϕ_k is defined to reflect keyword occurrence in a binary way:

$$\phi_k(p_i) = \begin{cases} 1 & \text{if } p_i \text{ contains at least one keyword} \\ 0 & \text{otherwise} \end{cases}$$

Function ϕ_c reflects citations. It is the sum of all scores of the cited posts:

$$\phi_c(p_i) = \sum_{x \in C_i} s(x) \qquad C_i = \{\text{posts cited by } p_i\}$$

Function ϕ_f reflects following of the previous post:

$$\phi_f(p_i) = s(p_{i-1})$$

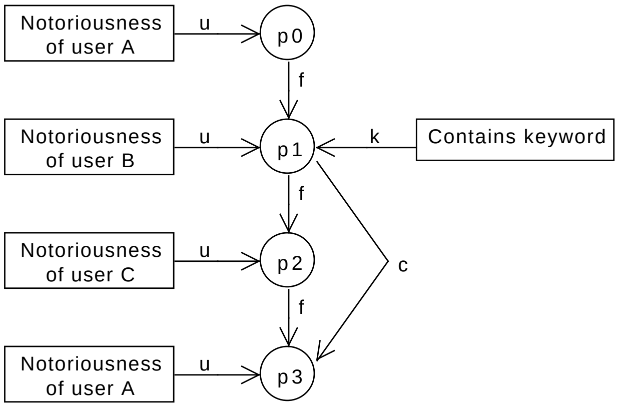

Figure 1: Intuitive graph-based visualization of an example thread. The circular nodes denote posts, the squared nodes denote features contributing relevance to the posts. Posts also inherit fractions of relevance scores from other posts by following them or citing them.

Function ϕ_u reflects the user behavior or "notoriousness" of a user. It is defined as the fraction of keyword-containing posts of that user, calculated from the whole corpus.[1]

$$\phi_u(p_i) = \frac{\#\text{ of keyword-containing posts of the author of } p_i}{\#\text{ of posts of the author of } p_i}$$

Figure 1 gives a visual example of the model and how the relevance scores are determined. Post 0 only draws relevance from the user behaviour edge. Post 1 has multiple sources of relevance, because it follows Post 0 and also contains a keyword. Post 3 cites Post 1 and thus gains additional relevance, which is proportional to the relevance score of Post 1. Thus, relevance propagates from node to node in a top-down way.

[1]This approach to modeling user behavior is rather simplistic and does not take into account that user behaviour might change over time. Future work may try to introduce an adaptive modeling of user behavior.

Finding Optimal Parameter Values

The regression model requires five parameters: The threshold t and the feature weights $k, c, f, u \in [0; 1]$. Because there is no appropriate way to set those parameters upfront, a Machine Learning technique has to be employed. More specifically, in a supervised learning setting, labeled posts shall be used to train the model.

Because relevance propagates through the graph, the relevance score of an individual posts depends on the scores of other nodes. Developing a mathematically exhaustive theory on the optimization of this model is non-trivial. Here, an alternative simplistic approach is chosen: A metaheuristic Evolutionary Algorithm [1][3]. This family of optimization algorithms has the great advantage of requiring only a very little understanding of the problem. Because it is defined what a possible solution is and how the fitness of such a solution can be determined, an Evolutionary Algorithm can be employed to optimize the parameters.

As a measure of fitness, the Matthews Correlation Coefficient (MCC) [4] is chosen. The (main) reasons are that the MCC is a common measure of success in binary classifications and that it is robust to biased samples. The proposed model does not only need to be trained, but the trained model also needs to be evaluated. Training and evaluating, however, can not be performed on the same data set, because this would result in overfitting. Therefore, the annotated data set needs to be divided into separate subsamples for training and testing. To achieve this, the well-established method of K-Fold Cross Validation [2] is employed and the parameter k is set to 10, which is a decision based on convention.

In order to justify the model complexity and prove the validity of the assumptions, the complex model needs to be compared against a baseline. To do so, two reduced models are inferred by removing some of the aforementioned features and parameters. The simplest baseline model utilizes keyword occurrence only. A more advanced baseline model utilizes keyword occurrence, citations, and post following as features, but no user notoriousness.

Algorithm Evaluation

First, we defined a set of 11 CCSVI-related keywords that include spelling variants:

- CCSVI
- CCVI
- Zamboni

- Stent
- Dilatation
- Chronic cerebrospinal venous insufficiency
- Chronic cerebro spinal venous insufficiency
- Chronic cerebro-spinal venous insufficiency
- Chronische Cerebro-Spinale Venöse Insuffizienz
- Chronische Cerebro Spinale Venöse Insuffizienz
- Chronische CerebroSpinale Venöse Insuffizienz

Based on these keywords, we identified partially relevant threads, i.e. those with at least 1 keyword-containing post. We then selected 51 partly relevant threads randomly, which contained 1348 posts. These posts were annotated manually. In the following paragraphs, the results of the three models being trained and evaluated using 10-Fold Cross Validation will be discussed. To do so, mean, standard deviation, minimum, and maximum of each parameter and measure of success are shown because the concrete values vary within the 10 rounds.

The first model is called the $t-k-model$, because it has a threshold t, but the only feature of a post is keyword occurrence (attributing a value of k). Table 1 shows that on average, parameter t has a value of 0.397 and parameter k has a value of 0.837. This is a plausible result of the heuristic training, because it means, that every post containing a keyword gets assigned a relevance score larger than the threshold and will thus be labeled relevant. The MCC value is not very high on average. The same holds true for the F_2-Measure.

The second model is called the $t-k-c-f-model$, because the features citations c and post following f are included additionally. The inclusion of these features raises the MCC on average by 0.099, as seen in Table 2. Parameter f has a value of 0.709 on average, which indicates that post following plays an important role in determining whether a post is relevant. The citation parameter c though has a much smaller value. This indicates that the citing of other posts seems to be a less important structural feature.

The third model is called the $t-k-c-f-u-model$, because it includes user notoriousness as an additional feature. Thus, all features discussed in Section are included in this model. Table 3 shows another slight increase in MCC and F_2-Measure values. Interestingly, the user notoriousness parameter u has a value of 0.556, which

Variable	Value			
	Mean	Std. Deviation	Minimum	Maximum
MCC	**0.558**	0.097	0.430	0.760
F_2-Measure	**0.523**	0.077	0.390	0.682
t	0.397	0.184	0.010	0.646
k	0.837	0.188	0.432	1.000

Table 1: Results of the 10-Fold Cross Validation using the tk-model.

Variable	Value			
	Mean	Std. Deviation	Minimum	Maximum
MCC	**0.657**	0.120	0.492	0.860
F_2-Measure	**0.822**	0.073	0.702	0.916
t	0.417	0.082	0.237	0.561
k	0.752	0.113	0.566	0.858
c	0.089	0.041	0.017	0.148
f	0.709	0.065	0.599	0.828

Table 2: Results of the 10-Fold Cross Validation using the tkcf-model.

shows, that some kind of repetitive behavior of users does exist and that this information can be useful in determining relevance. It also shows, that all discussed features are of value when determining relevance. Thus, the most complex model is used for finding relevant posts. Averaged parameter values over the 10 rounds are used for the final Information Retrieval.

Variable	Value			
	Mean	Std. Deviation	Minimum	Maximum
MCC	**0.699**	0.102	0.578	0.893
F_2-Measure	**0.844**	0.061	0.746	0.928
t	0.435	0.114	0.152	0.591
k	0.927	0.070	0.760	1.000
c	**0.067**	0.019	0.034	0.089
f	0.612	0.092	0.358	0.693
u	**0.556**	0.128	0.395	0.860

Table 3: Results of the 10-Fold Cross Validation using the tkcfu-model.

Bibliography

[1] E. Alba and C. Cotta. Evolutionary algorithms. *Handbook of Bioinspired Algorithms and Applications. Boca Raton: Chapman and Hall/CRC*, 2006.

[2] T. Hastie, R. Tibshirani, and J. Friedman. *The Elements of Statistical Learning.* Springer, Jan. 2009.

[3] K. A. D. Jong. *Evolutionary computation: a unified approach.* MIT Press, Cambridge, Massachusetts, Feb. 2006.

[4] B. W. Matthews. Comparison of the predicted and observed secondary structure of t4 phage lysozyme. *Biochimica et biophysica acta*, 405(2):442, 1975.

[5] F. Sudau. Analysis of controversial debates in online fora - a showcase analysis of the CCSVI discussion in the DMSG layperson forum. *Master's Thesis in Applied Computer Science at the Institute of Computer Science, ZAI-MSC-2013-04, ISSN 1612-6793, Georg-August-University of Göttingen.*, Apr. 2013.
