# Supplementary material for: Sources of Information and Behavioral Patterns in Online Health Forums: Observational Study
Source: J Med Internet Res. 2014 Jan 14;16(1):e10. doi: 10.2196/jmir.2875 (PMC3958625; doi:10.2196/jmir.2875)
Supplement: Supplementary file 2 [file jmir_v16i1e10_app2.pdf]

K-Means Clustering

Here, we want to discuss the algorithm used for the user pattern discovery. Generally speaking, clustering describes the task of grouping objects together with the intention of having similar objects in the same group and dissimilar objects across groups. Thus, clustering is a form of exploratory data analysis suited for the discovery of previously unknown patterns. Clustering algorithms typically take points in a hyperspace as input and produce groups of them as output. A human analyst can then take a look at descriptions of the groups and identify, which patterns distinguish the members of one group from those of other groups.

We proceeded as follows:

1. We represented every user by a data point in euclidean $\mathbb{R}^d$ by finding appropriate numerical features and normalizing them. This means our n users were represented by $x_1, x_2, \ldots, x_n \in \mathbb{R}^d$.

2. We performed repeated K-Means clusterings with different values of K (the number of clusters). Depending on the resulting cluster size distributions and internal evaluation metrics, we chose the best performing K. We used the Davies Bouldin Index [2], the Dunn Index [3] and a variant of the Dunn Index based on average linkage as evaluation metrics and looked for sharp increases of the metric (in the case of the Dunn indices) or decreases (in the case of Davies Bouldin).

3. We interpreted summary statistics of the clusters resulting form the best value of K in order to give names to the clusters.

The K-Means is a well known and very traditional algorithm first proposed in [4]. It is efficient and known for producing quite good results despite the heuristic nature of the algorithm. The algorithm uses centroids as cluster-defining entities and proceeds as described in Algorithm 1.

The abstract steps mentioned in Algorithm 1 describe the following:

Initialize Originally, the K initial centroids were placed randomly in the feature space. This, however, left the results open to pure chance and a bad placement of the original centroids could lead to an unwanted final result. Therefore, the deterministic

Initialize: Place k initial centroids $c_1, c_2, \ldots, c_k$ somewhere in the vector space;
repeat
  Assign each data point x_i to the nearest centroid c;
  Update each centroid c_i to be the centroid of all data points assigned to it;
until *convergence criterion is reached*;

Algorithm 1: The K-Means algorithm on a high level of abstraction.

initialization step proposed in [1] is used in this work. The proposed alternative is of heuristic nature and is expected to produce better results on average than the random initialization. It proceeds as follows:

- The two data points with the greatest distance in between them are selected as c_1 and c_2:

$$c_1 = x_j, c_2 = x_k : \|x_j - x_k\| \geq \|x_l - x_m\| \forall x_l, x_m \in X.$$

  The euclidean distance between two points p and q in a d-dimensional space is defined as

$$\|p - q\| = \sqrt{(p_1 - q_1)^2 + (p_2 - q_2)^2 + \ldots + (p_d - q_d)^2}.$$

- Every remaining centroid is placed in an iterative way one after another. When the *nth* centroid c_n needs to be placed in the vector space, the data point with the largest minimum distance to all previous centroids is chosen. If R denotes the set of every data point that has not been used as a centroid yet, the assignment of the *nth* centroid can be formulated mathematically as

$$c_n = \arg\max_{x \in R}(\min(\{\|x - c_1\|, \|x - c_2\|, \ldots, \|x - c_{n-1}\|\}))$$
$$R = \{x_1, x_2, \ldots, x_n\} \setminus \{c_1, c_2, \ldots, c_{n-1}\}.$$

Assign Assigning a data point x to the closest centroid means finding the centroid with the minimum euclidean distance to the data point. The used distance measure is the same as the one used in the initialization step.

Update Each centroid is assigned the arithmetic mean of all the data points assigned to it. Let S_i denote the set of all data points assigned to c_i. The assignment can then be expressed as

$$c_i = \frac{1}{|S_i|} \cdot \sum_{x_i \in S_i} x_i.$$

Convergence criterion In this case, total convergence is used as a criterion: The algorithm stops, when no data point was re-assigned to another centroid in the current iteration.

Bibliography

[1] J. Couto. Kernel k-means for categorical data. *Advances in Intelligent Data Analysis VI - 6th international Symposium on Intelligent Data Analysis, IDA 2005 Proceedings*, pages 46–56, 2005.

[2] D. L. Davies and D. W. Bouldin. A cluster separation measure. *IEEE Transactions on Pattern Analysis and Machine Intelligence*, (2):224–227, 1979.

[3] J. C. Dunn. A fuzzy relative of the ISODATA process and its use in detecting compact well-separated clusters. *Journal of Cybernetics*, 3(3):32–57, 1973.

[4] J. MacQueen. Some methods for classification and analysis of multivariate observations. In *Proceedings of the 5th Berkeley symposium on mathematical statistics and probability*, volume 1, pages 281–297, 1967.
