# Supplementary material for: Sources of Information and Behavioral Patterns in Online Health Forums: Observational Study
Source: J Med Internet Res. 2014 Jan 14;16(1):e10. doi: 10.2196/jmir.2875 (PMC3958625; doi:10.2196/jmir.2875)
Supplement: Supplementary file 3 [file jmir_v16i1e10_app3.pdf]

Table 1: Aggregated number of references posted to each domain class from each cluster. Unnormalized values.

	Users of Uncommon Sources	Organization Followers	Seekers of Health Care	Social Media Fans	Homepage Promoters	Balanced Source Users
commerce	9	2	1	18	1	14
news	16	13	2	80	10	**64**
organization	29	**54**	2	110	5	**50**
other	**44**	1	0	44	0	22
personal	11	5	0	21	**46**	8
scientific	12	10	1	65	0	**53**
social	31	6	6	**540**	14	**76**
healthcare providers	10	0	**16**	32	1	11

Table 2: Arithmetic mean and (standard deviation) of the contribution behavior feature values for each cluster. Unnormalized Values.

	Soph. Contr.	CCSVI Act.	CCSVI-F. Resp.	Short-l. CCSVI-Sp.	H.A.R.P.	Avg. Users
Average message length	**1293.9** (387.7)	380.5 (168.6)	496.0 (268.6)	**271.0** (097.7)	510.8 (338.4)	448.0 (226.6)
Average number of references per post	**0.917** (0.280)	**0.533** (0.254)	0.150 (0.141)	0.050 (0.087)	0.202 (0.159)	0.185 (0.178)
Average number of posts per day	0.301 (0.272)	0.560 (0.537)	**0.246** (0.222)	**5.667** (1.958)	**3.971** (1.907)	0.593 (0.580)
Average number of threads per day	0.061 (0.038)	0.255 (0.217)	0.147 (0.122)	0.958 (0.298)	**1.339** (0.418)	0.239 (0.201)
Days Active	467.0 (325.1)	515.2 (447.1)	292.0 (293.0)	**1.750** (0.829)	554.1 (441.2)	793.1 (427.5)
Fraction of posts that were cited	0.131 (0.111)	0.164 (0.150)	0.210 (0.146)	0.330 (0.196)	0.202 (0.059)	0.149 (0.103)
Fraction of relevant posts	0.264 (0.213)	**0.379** (0.159)	0.534 (0.156)	**0.839** (0.278)	0.099 (0.069)	0.104 (0.092)
Fraction of initiated threads	0.309 (0.257)	**0.458** (0.130)	**0.089** (0.097)	0.000 (0.000)	**0.064** (0.108)	0.117 (0.089)
Coverage of users in relevant parts per post	0.351 (0.236)	**0.500** (0.377)	**1.143** (0.765)	**1.078** (0.517)	0.061 (0.074)	0.178 (0.168)
