# Supplementary material for: Sources of Information and Behavioral Patterns in Online Health Forums: Observational Study
Source: J Med Internet Res. 2014 Jan 14;16(1):e10. doi: 10.2196/jmir.2875 (PMC3958625; doi:10.2196/jmir.2875)
Supplement: Supplementary file 4 [file jmir_v16i1e10_app4.pdf]

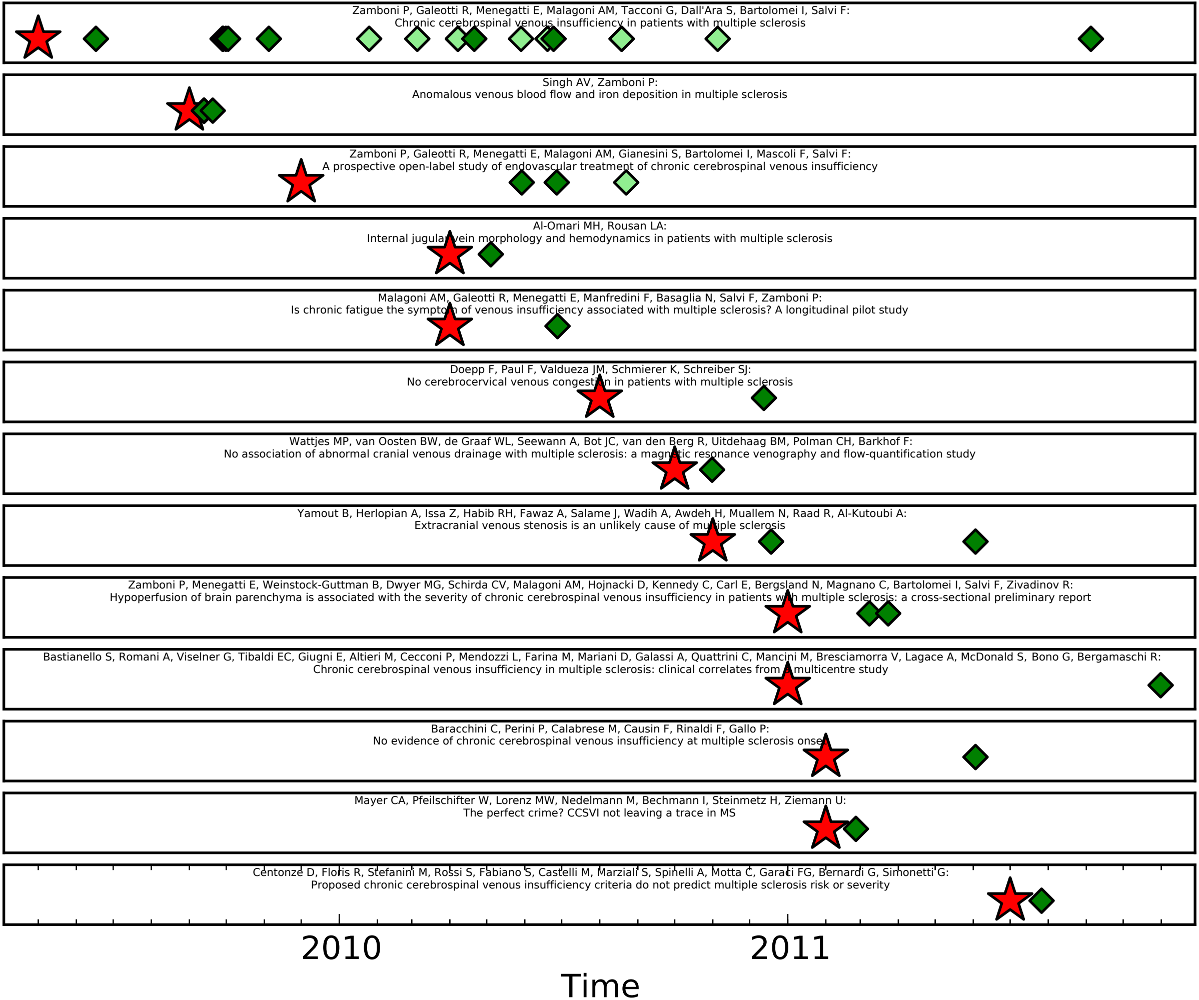

Each publication is shown in a separate area where the red star indicates the publication date and the green diamonds show dates where links to the publication were posted. Light green diamonds indicate indirect references.
